# Met receptor-induced Grb2 or Shc signals both promote transformation of intestinal epithelial cells, albeit they are required for distinct oncogenic functions

**DOI:** 10.1186/1471-2407-14-240

**Published:** 2014-04-04

**Authors:** Véronique Pomerleau, Mélissa Landry, Jimmy Bernier, Pierre H Vachon, Caroline Saucier

**Affiliations:** 1 Department of Anatomy and Cell Biology, Faculty of Medicine and Health Sciences, Université de Sherbrooke, 3201, rue Jean-Mignault, Sherbrooke, Quebec J1E 4K8, Canada

**Keywords:** Receptor tyrosine kinase, Grb2, Shc, Met, Intestinal epithelial cell, Transformation, Proliferation, Anoikis, MAPK, PI3K, Colorectal cancer

## Abstract

**Background:**

Deregulation of receptor tyrosine kinases (RTK) contributes to the initiation and progression of intestinal-derived epithelial cancers, including colorectal cancer (CRC). However, the roles of the proximal signaling molecules engaged by RTKs in different oncogenic functions of CRC remain unclear.

**Methods:**

Herein, the functional impact of expressing variant forms of the oncogenic Met receptor (Tpr-Met) that selectively recruit the adaptor proteins Grb2 or Shc was investigated in a model derived from normal intestinal epithelial cells (IEC-6). An RNA interference (RNAi) approach was used to define the requirement of Grb2 or Shc in Tpr-Met-transformed IEC-6 cells. Since Grb2 and Shc couple RTKs to the activation of the Ras/MEK/Erk and PI3K/Akt pathways, Erk and Akt phosphorylation/activation states were monitored in transformed IEC-6 cells, and a pharmacological approach was employed to provide insights into the roles of these pathways in oncogenic processes evoked by activated Met, and downstream of Grb2 and Shc.

**Results:**

We show, for the first time, that constitutive activation of either Grb2 or Shc signals in IEC-6 cells, promotes morphological transformation associated with down-regulation of E-cadherin, as well as increased cell growth, loss of growth contact inhibition, anchorage-independent growth, and resistance to serum deprivation and anoikis. Oncogenic activation of Met was revealed to induce morphological transformation, E-cadherin down-regulation, and protection against anoikis by mechanisms dependent on Grb2, while Shc was shown to be partly required for enhanced cell growth. The coupling of activated Met to the Ras/MEK/Erk and PI3K/Akt pathways, and the sustained engagement of Grb2 or Shc in IECs, was shown to trigger negative feedback, limiting the extent of activation of these pathways. Nonetheless, morphological alterations and E-cadherin down-regulation induced by the oncogenic Tpr-Met, and by Grb2 or Shc signals, were blocked by MEK, but not PI3K, inhibitors while the enhanced growth and resistance to anoikis induced by Tpr-Met were nearly abolished by co-treatment with both inhibitors.

**Conclusion:**

Overall, these results identify Grb2 and Shc as central signaling effectors of Met-driven progression of intestinal epithelial-derived cancers. Notably, they suggest that Grb2 may represent a promising target for the design of novel CRC therapies.

## Background

When activated, cell surface growth factor receptor tyrosine kinases (RTK) become phosphorylated at a number of tyrosine (Tyr) residues. Many of those located within the cytoplasmic tail of the receptor create binding sites for proteins containing Src homology 2 (SH2) and phosphotyrosine-binding (PTB) domains, which recognize phospho-Tyr residues within the context of specific adjacent amino acids. Proteins recruited to RTKs include enzymes, such as phospholipase Cγ (PLCγ) and phosphatidylinositol 3-kinase (PI3K); and adaptor proteins, including growth factor receptor-bound protein-2 (Grb2) and Src-homology collagen (Shc) proteins. These latter proteins contain multiple protein-protein interactions motifs. The resulting complex relays and amplifies an exquisitely fine-tuned regulation of multiple downstream signaling events, which depending on cellular context, mediate specific biological responses (reviewed in [1]). This regulation is perturbed in cancers, including those of intestinal epithelial cell (IEC) origin such as colorectal cancer (CRC).

Deregulation of RTKs in CRC commonly involves the over-expression of the receptor and/or its ligand. As illustrated by the Met/hepatocyte growth factor (HGF) and epidermal growth factor receptor (EGFR) signaling axis, this dysregulation often takes place at the earliest stages of the disease and it is observed in virtually all metastatic CRC (reviewed in [2-4]). Ligand or receptor deregulation may result in a lower threshold for growth factor stimulation, autocrine/paracrine ligand-receptor activation loops, and even ligand-independent constitutive receptor activation. Regardless of the mechanisms of RTK oncogenic activation, the outcome is the loss of the normally fine-tuned regulation of downstream signaling, which may ultimately contribute to the acquisition of cancer properties. Notably, its has been shown that the expression of constitutively activated forms of the Met receptor in non-transformed IECs promoted morphological transformation; enhanced proliferation rate; induced loss of both growth contact inhibition and anchorage-dependent growth; and increased *in vivo* angiogenic, tumorigenic, and metastatic capacities [5,6].

Studies performed predominantly in fibroblast and breast cancer cell models have revealed that Grb2 and Shc adaptor proteins are among the signaling proteins that, upon recruitment by activated RTKs, mediate events directly linked to the initiation and progression of cancer [7-12]. Many RTKs interact directly with Grb2, some rely on Shc family adaptors to recruit Grb2, and others do both [1]. While direct Grb2/RTK interactions involve binding of the Grb2 SH2 domain to pYXNX motifs, Shc proteins interact with RTKs primarily through the binding of their N-terminal PTB domain to NPXpY motifs. The latter results in phosphorylation of Tyr residues within the Shc central collagen-homology domain 1 (CH1). These phosphorylated tyrosine residues constitute consensus-binding sites for the Grb2 SH2 domain, thus allowing Shc to engage Grb2-driven signaling pathways (reviewed in [13]). The best-characterized role of the two adaptor proteins, Grb2 and Shc, is to link RTKs to the activation of the Ras/Raf/MEK/Erk mitogenic (Ras/MAPK) pathway. The constitutive association of the N-terminal Grb2 SH3 domain with the Ras guanine nucleotide exchange factor, Son of Sevenless (SOS) constitutes one component of this connection [1]. Interaction of the C-terminal Grb2 SH3 domain with Grb2-associated binding (Gab) scaffold protein family members couples RTKs to the PI3K/Akt survival pathway and to the Ras/MAPK cascade by an alternate route [14]. As such, the recruitment of Grb2 or Shc to RTKs has been shown to promote biologically redundant processes [7,8,15,16]. However, Shc proteins interact with diverse signaling molecules in addition to Grb2, thereby engage Grb2-independent pathways and biological functions [9-13,17-19].

Although the deregulation of RTKs is widely considered to be a major determinant in the progression of CRC, the specific contributions of the proximal signaling molecules engaged by these receptors in CRC remain virtually unexplored. Herein, we report the exploitation of well-characterized adaptor-specific RTK docking variants derived from the oncogenic Met receptor, Tpr-Met [8,9,15,16,20], with shRNA and pharmacological interference approaches to define, for the first time, the cancer properties associated with early neoplastic transformation of IECs, induced upon oncogenic mediated activation of either Grb2 or Shc signaling.

## Methods

### Antibodies and reagents

The Met polyclonal antibody, kindly provided by Dr. Morag Park (McGill University, Montreal, QC, Canada), was raised against an epitope in the C-terminal region of human Met, distinct from those altered in the variants (Additional file 1) [8,21]. The Phospho-Tyr (p-Tyr100), phospho-Akt (Ser473), and phospho-Erk1/2 (p44/42MAPK, Thr202/Tyr204) antibodies were obtained from Cell Signaling Technology (Danvers, MA, USA). The pan-Shc and phospho-Tyr Shc (Tyr239/240) antibodies, that recognize the p66, p52, and p46 isoforms of ShcA, and the Erk2 antibody were obtained from Santa Cruz Biotechnology (Santa Cruz, CA, USA). The α-tubulin and β-actin antibodies were from Sigma-Aldrich Canada Ltd (Oakville, ON, Canada). The Grb2 and E-cadherin antibodies were purchased from BD Transduction Labs (Lexington, KY, USA). The MEK1/2 and PI3K inhibitors, U0126 and LY294002, were purchased from Cell Signaling Technology, while the MEK1/2 inhibitors AZD6244 and PD184352 were obtained from Selleck Chemicals (Houston, TX, USA).

### Grb2- and Shc-specific docking Tpr-Met variants and shRNAs

As depicted in Additional file 1, the RTK-derived docking oncoproteins consist of an oncogenic form of the Met receptor, Tpr-Met, in which the multi-substrate binding region is replaced with a motif selective for the recruitment of a single signaling protein, found in other RTKs [8]. The Grb2-specific Tpr-Met variant (TM-Grb2) contains the Grb2 binding site derived from EGFR. Docking variants specific for the recruitment of Shc include either the high affinity Shc binding motif from the TrkA receptor (TM-Shc1), or the lower affinity motif from EGFR (TM-Shc2). The generation of these specific docking Tpr-Met variants, and their insertion into the pLXSN retroviral expression vector were previously described [8]. For stable silencing of Grb2 and Shc in cells, a short hairpin RNA (shRNA) lentiviral vector-based approach was used. The design of the pLenti6-U6 construct for the stable transduction of shRNAs and the blasticidin S resistance gene has been described previously [22]. Clontech shRNA Sequence Designer tool was used to create an shRNA containing nucleotide sequences that target rat Grb2 mRNA [23], the three ShcA isoforms [24], or a non-targeting control sequence [shGrb2 (NM_030846): 5′-GATGTACAGCACTTCAAGGTG-3′; shShc (NM_053517): 5′-CTACTTGGTTCGGTACATGGG-3′; shCTRL: 5′-GCTTTCCCGTCACGCGTACCT-3′].

### Cell culture

Normal rat intestinal epithelial crypt cells, IEC-6, provided by Dr. A. Quaroni (Cornell University, Ithaca, NY), were maintained in DMEM (Wisent, St-Bruno, QC, Canada) containing 10% fetal bovine serum (FBS, Life Technologies, Burlington, ON, Canada) and 50 μg/mL gentamicin (Wisent). The IEC-6 cells were validated as non-transformed, and displaying normal features including an epithelioid morphology with sparse microvilli and E-cadherin cell-cell interactions, and a rate of cell division estimated by growth curve analysis that is typical of normal undifferentiated IECs [5,22,25]. Stable populations of IEC-6 cells expressing TM-Grb2, -Shc1, or -Shc2 (TM-Grb2-IEC-6, TM-Shc1-IEC-6, and TM-Shc2-IEC-6 cells, respectively) were expanded, following retroviral infection, from a pool of at least 50 neomycin-resistant colonies, as described for the generation of IEC-6 cells transformed with unmodified Tpr-Met (Tpr-Met-IEC-6 cells) [5]. For each experiment, populations of TM-Grb2-IEC-6, TM-Shc1-IEC-6, or TM-Shc2-IEC-6 cells were compared with the previously characterized sham-infected IEC-6 (Control-IEC-6) and Tpr-Met-IEC-6 cells [5], each having been passaged a comparable number of times (11–25 ± 2). The binding specificity of these Tpr-Met variants was extensively validated in earlier studies [8,9,15,16,20,26], and further confirmed in IEC-6 cells (Additional file 1). Stable knockdown of Grb2 or Shc expression in Tpr-Met-IEC-6 cells was achieved by lentiviral-mediated transduction of appropriate shRNAs. Production of replication-deficient lentiviruses in HEK 293 T cells and infection of Tpr-Met-IEC-6 cells were performed as previously described [22]. Stable populations of Tpr-Met-IEC-6 cells expressing the shRNA against Grb2 or ShcA (TM-shGrb2 or TM-shShc cells) were selected, and thereafter maintained with blasticidin S HCl (5 μg/mL). A control Tpr-Met-IEC-6 cell population expressing a non-targeting shRNA was likewise generated (TM-shCTRL).

### Immunoprecipitation and immunoblotting

Total cell lysate (TCL) preparation, SDS-PAGE, immunoprecipitation (IP), and immunoblot (IB) analysis methods have previously been described [8,9]. Primary antibodies were used at a concentration of 1:1000, with the exception of Akt and P-Akt (1:500), Erk2 (1:5000), P-Erk (1:2500), E-cadherin (1:5000), and α-tubulin and β-actin (1:10000). Secondary antibodies were used at a concentration of 1:10000. Proteins were visualized by enhanced chemiluminescence (ECL, GE Healthcare, Baie d’Urfé, QC, Canada). Unless otherwise indicated, biochemical analyses were performed at least three times with independent lysate preparations from cells that had been serum-starved overnight.

### Semi-quantitative and quantitative RT-PCR

Total RNA from serum-starved cells was extracted using TRIzol (Life Technologies) or RiboZol™ RNA Extraction Reagent (Amresco, Solon, OH), following the manufacturer’s protocols. The RNA integrity was assessed with an Agilent 2100 Bioanalyzer (Agilent Technologies, Santa Clara, CA) and quantitation was performed using a Nanodrop spectrometer (Thermo Fisher). Reverse transcription was performed on DNase I-treated RNA (Amplification grade, Life Technologies) with Omniscript Reverse Transcriptase (Qiagen). Semi-quantitative PCR reactions were carried out using TOPTaq (Qiagen), according to the manufacturer’s protocol. Resulting PCR products were then analyzed on a 2% agarose gel. Quantitative real-time PCR analyses were performed by the RNomics Platform at the Université de Sherbrooke (Sherbrooke, QC, Canada). The sequence of the primers used is listed in Additional file 2.

### Cell-count, focus formation, soft agar growth, and anoikis assays

For cell-count assays, cells were seeded at a density of 2.5 × 10^4^/well in 6-well plates, and then counted daily. For focus formation assays, 200 cells of the experimental populations were seeded in each well of 6-well plates, with 5 × 10^5^ parental IEC-6 cells forming a monolayer in each. After 10–15 days, the foci were photographed, fixed with 10% formalin buffer and stained with Giemsa for counting. Soft agar growth assays were conducted with 5000 cells per well, embedded in Noble Agar (Difco, BD Transduction Labs, Lexington, KY, USA). Colonies present after approximately two weeks were photographed and counted. For anoikis assays, cells were seeded in serum-free DMEM (or Opti-MEM) at a density of 1.25 × 10^5^ cells/well in 24-well plates that were pre-coated (or not) with polyHEMA [poly-(2-hydroxyethyl methacrylate); Sigma]. After 18 hours, cell viability was determined by XTT assays [27]. The proportion of viable cells in polyHEMA-coated wells (suspension), relative to those seeded in non-coated wells (adherent), was calculated. Growth curves and bar graphs were generated using Prism v6.0c (GraphPad software, San Diego, CA, USA). Unless otherwise indicated, results are expressed as the mean ± S.E.M. from three independent experiments, each performed in triplicate. Statistical significance was determined by ANOVA analysis (* indicates a p-value < 0.05; **, p < 0.01; ***, p < 0.001; and ****, p < 0.0001).

### Ethics statement

The research carried out did not involve any human subjects.

## Results

### Oncogenic engagement of Grb2 or Shc signaling promotes morphological transformation in normal IECs and reduces E-cadherin expression

In contrast with many other RTKs, the ability of the Met receptor to recruit signaling proteins, and thus its biological activities, is dependent on two phospho-Tyr residues located within its C-terminus (Y1349/Y1356 in Met; Y482/Y489 in Tpr-Met [28-30]). This unique characteristic has been exploited to generate Met receptor variants capable of binding exclusively to a single RTK-proximal signaling protein [8]. These well-characterized docking-specific Met variants consist of the cytosolic oncogenic form of the Met receptor, Tpr-Met, in which the multi-substrate binding region has been replaced with a motif selective for the recruitment of a single signaling protein, found in other RTKs (Additional file 1 and [8]). To determine whether the selective oncogenic engagement of either the Grb2 or the Shc adaptor protein was sufficient to induce transformation of IECs, we generated populations of IEC-6 cells [25] expressing Tpr-Met variants that specifically bind to either the Grb2 (TM-Grb2-IEC-6 cells) or Shc (TM-Shc1-IEC-6 and TM-Shc2-IEC-6 cells, respectively) adaptor proteins, through retroviral infection. For each experiment, these cells were compared with the previously characterized untransformed sham-infected IEC-6 (Control-IEC-6) cells or those transformed by unmodified Tpr-Met (Tpr-Met-IEC-6) [5].

**Figure 1:**
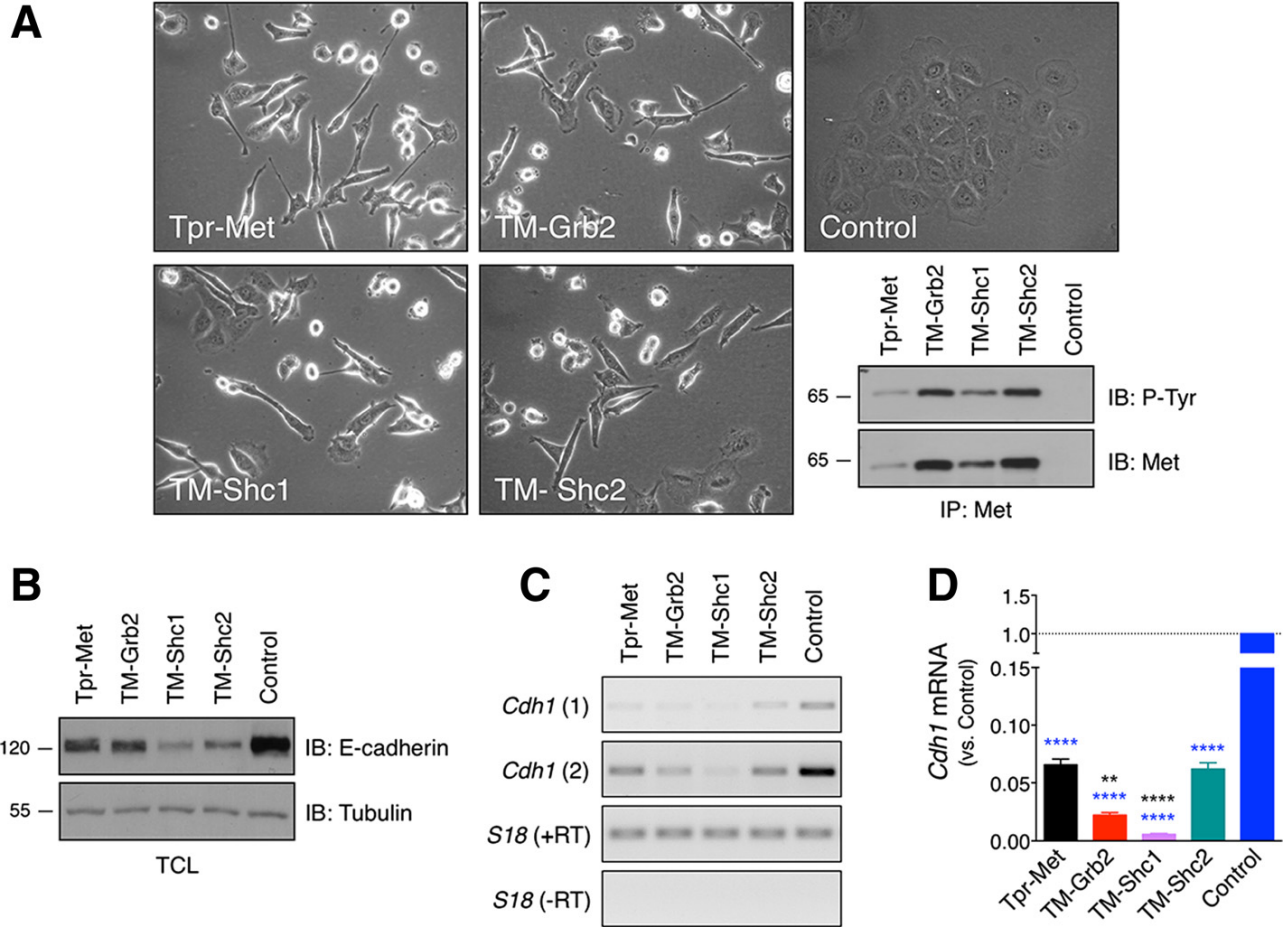
**TM-Grb2, TM-Shc1 and TM-Shc2 oncoproteins promote morphological transformation and E-cadherin down-regulation in IEC-6 cells. (A)** Photographs show the typical morphology of IEC-6 cells expressing, or not (Control), the docking-specific TM-Grb2, TM-Shc1 or TM-Shc2, or the oncogenic Met receptor, Tpr-Met. Expression and phosphorylation levels of these oncoproteins in IEC-6 cell lysates were assessed by immunoblot (IB) analyses following immunoprecipitation (IP) with a Met antibody. **(B)** E-cadherin protein levels were determined by IB analyses of total cell lysate (TCL) prepared from the indicated serum-starved cells. Tubulin protein levels provide a loading control. **(C)** E-cadherin mRNA expression (*Cdh1*) was evaluated by semi-quantitative RT-PCR assays performed with total RNA prepared from the indicated serum-starved cells. The analyses were carried out with two sets of primers designed to amplify distinct regions of the rat *Cdh1* mRNA. The mRNA encoding for the *S18* ribosomal protein is shown as a loading control. Parallel PCR reactions without the RT enzyme indicate that the amplified products did not arise from genomic DNA contamination. **(D)** Relative mRNA expression levels of *Cdh1* were analyzed by quantitative real-time RT-PCR. These assays were carried out with the same RNA samples and sets of *Cdh1* primers. The bar graph shows the mean ± S.E.M. fold-change of *Cdh1* mRNA levels relative to Control-IEC-6 cells. The values are from three independent sample sets run in duplicate, normalized to *TATA-binding protein* (*TBP*), *pumilio RNA-binding family member 1* (*Pum1*) and *ribosomal protein L19* (*Rpl19*) expression. The primer sequences are listed in Additional file 2.

TM-Grb2-IEC-6, TM-Shc1-IEC-6, and TM-Shc2-IEC-6 cells were morphologically transformed to a similar extent relative to Control-IEC-6 cells, which grew in colonies and displayed typical normal epithelioid morphology (Figure 1A). Morphological changes induced in the TM-Grb2-IEC-6, TM-Shc1-IEC-6, and TM-Shc2-IEC-6 cells include an apparent breakdown of cell-cell contacts, cell dispersal, the acquisition of a fibroblast-like spindle-shaped morphology, and a more refractile appearance than the Control-IEC-6 cells. Cells expressing the Grb2 and Shc docking-specific oncoproteins also displayed many cell membrane protrusions typical of lamellipodia and invadopodia-like structures. The Tpr-Met variants exhibited comparable levels of Tyr phosphorylation in IEC-6 cells, relative to their expression levels (Figure 1A). They also demonstrated the predicted binding selectivity [8,9,15,16,20] (Additional file 1).

Since such morphological changes in epithelial cells are typically associated with down-regulation of E-cadherin, immunoblot (IB) analysis of the E-cadherin protein levels were performed [31]. Consistent with the previous characterization of Tpr-Met-IEC-6 cells [5], also presented herein, oncogenic activation of Grb2- and especially Shc-specific signals led to a dramatic decrease in E-cadherin protein levels relative to the Control-IEC-6 cells (Figure 1B). Likewise, a marked reduction in E-cadherin (*Cdh1*) mRNA levels was observed in these cells, as determined by semi-quantitative and quantitative real-time RT–PCR analyses (Figure 1C and D). Analysis of the expression profiles of E-cadherin transcriptional repressors in these IECs suggests that a combined up-regulation of *Snail2*, *Twist1,* or *Twist2*, but not of *Snail1* or *Zeb1*, may partly account for the E-cadherin repression induced by oncogenic Met-dependent signaling, including that one driven by Grb2 and Shc signals (Additional file 2). Overall, these results demonstrate that oncogenic activation of Grb2- and Shc-dependent signaling pathways, such as those activated by Tpr-Met, is sufficient to induce an epithelial-mesenchymal morphological-like transformation in normal IECs.

### Oncogenic engagement of Grb2 or Shc signaling enhances cell growth and loss of contact inhibition in IECs

**Figure 2:**
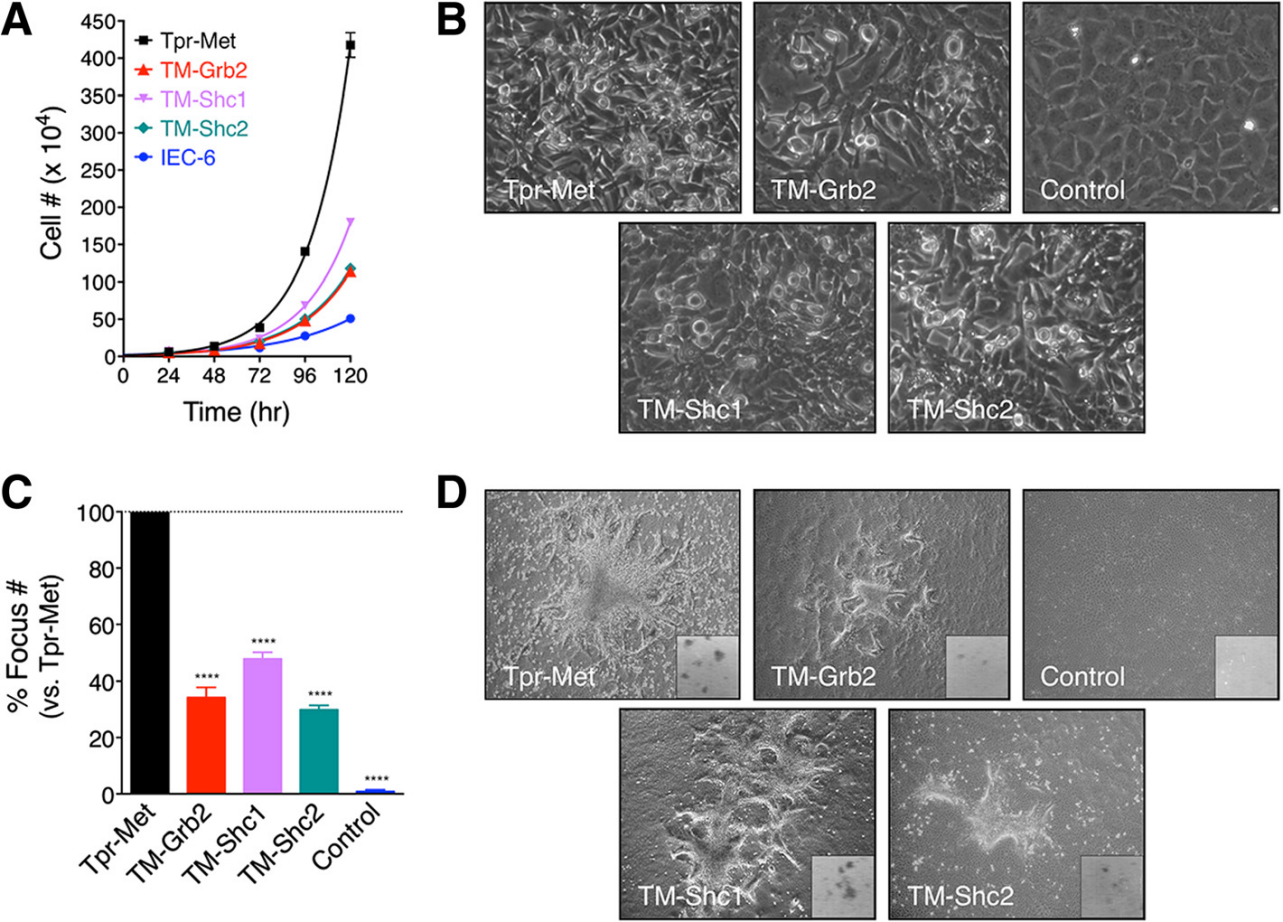
**TM-Grb2, TM-Shc1 and TM-Shc2 oncoproteins elicit cell growth and loss of contact inhibition of growth in IEC-6 cells. (A)** Representative growth curves of IEC-6 cells expressing, or not (Control), the indicated Tpr-Met-derived oncoproteins. The doubling times calculated in hours, from 3 independent experiments performed in triplicate, are for Tpr-Met-IEC-6: 15.4 ± 0.3; TM-Grb2-IEC-6: 21.5 ± 0.6; TM-Shc1-IEC-6: 18.3 ± 0.5; TM-Shc2-IEC-6: 20.9 ± 0.7 and Control-IEC-6: 29.5 ± 1.9. **(B)** Photographs show typical morphology of the cells cultured at high density in the presence of serum. **(C)** Focus formation assays were performed with the indicated IEC-6 cell populations. The bar graph shows the average number of foci over ~10 days counted on stained plates in 3 independent experiments performed in triplicate. Values are expressed as percentage foci ± S.E.M. of those formed by Tpr-Met-IEC-6 cells. **(D)** Photographs show representative morphology of the foci formed prior to formalin fixation and after Giemsa staining (small insets).

We next tested the oncogenic growth characteristics of IEC-6 cells transformed by the Tpr-Met variants. First, cell-counting assays were performed to determine the growth rate of each cell population. The TM-Grb2-IEC-6, TM-Shc1-IEC-6, and TM-Shc2-IEC-6 cells displayed more rapid growth than Control-IEC-6 cells, albeit to a lesser extent than Tpr-Met-IEC-6 cells (Figure 2A). Notably, TM-Shc1-IEC-6 cells exhibited accelerated growth relative to TM-Grb2-IEC-6 and TM-Shc2-IEC-6 cells, between which the doubling time did not differ significantly. The enhanced growth promoting activity of the TM-Shc1 relative to the TM-Shc2 correlates with the affinity of their respective Shc binding sites [8]. Typical of untransformed cells, IEC-6 cells ceased proliferation upon the establishment of cell-cell contacts [5,25]. As such, Control-IEC-6 cells formed a monolayer of well-organized epithelial cells upon reaching confluence (Figure 2B). In sharp contrast, each of the oncogenic transformed IEC-6 cell populations appeared highly disorganized and grew in multiple layers at confluence (Figure 2B). Thus, oncogenic specific activation of Grb2- or Shc-dependent signals permits IECs to bypass contact inhibition of growth. To validate this in a quantitative manner, focus formation assays were performed. As expected [5], Control-IEC-6 cells failed to form foci, whereas TM-Grb2-IEC-6, TM-Shc1-IEC-6, and TM-Shc2-IEC-6 cells displayed strong focus-forming capacities, though less than that of Tpr-Met-IEC-6 cells (Figure 2C and D). The number and size of foci formed by the TM-Shc1-IEC-6 cells were markedly greater than those of the TM-Grb2-IEC-6 or TM-Shc2-IEC-6 cells (Figure 2C and D), likely reflecting their accelerated growth rate (Figure 2A). These results indicate that the oncogenic engagement of Grb2- and Shc-dependent signals is sufficient to relieve contact inhibition of growth in IECs.

### Oncogenic engagement of Grb2 and Shc signaling induces anchorage-independent growth and anoikis resistance in IECs

**Figure 3:**
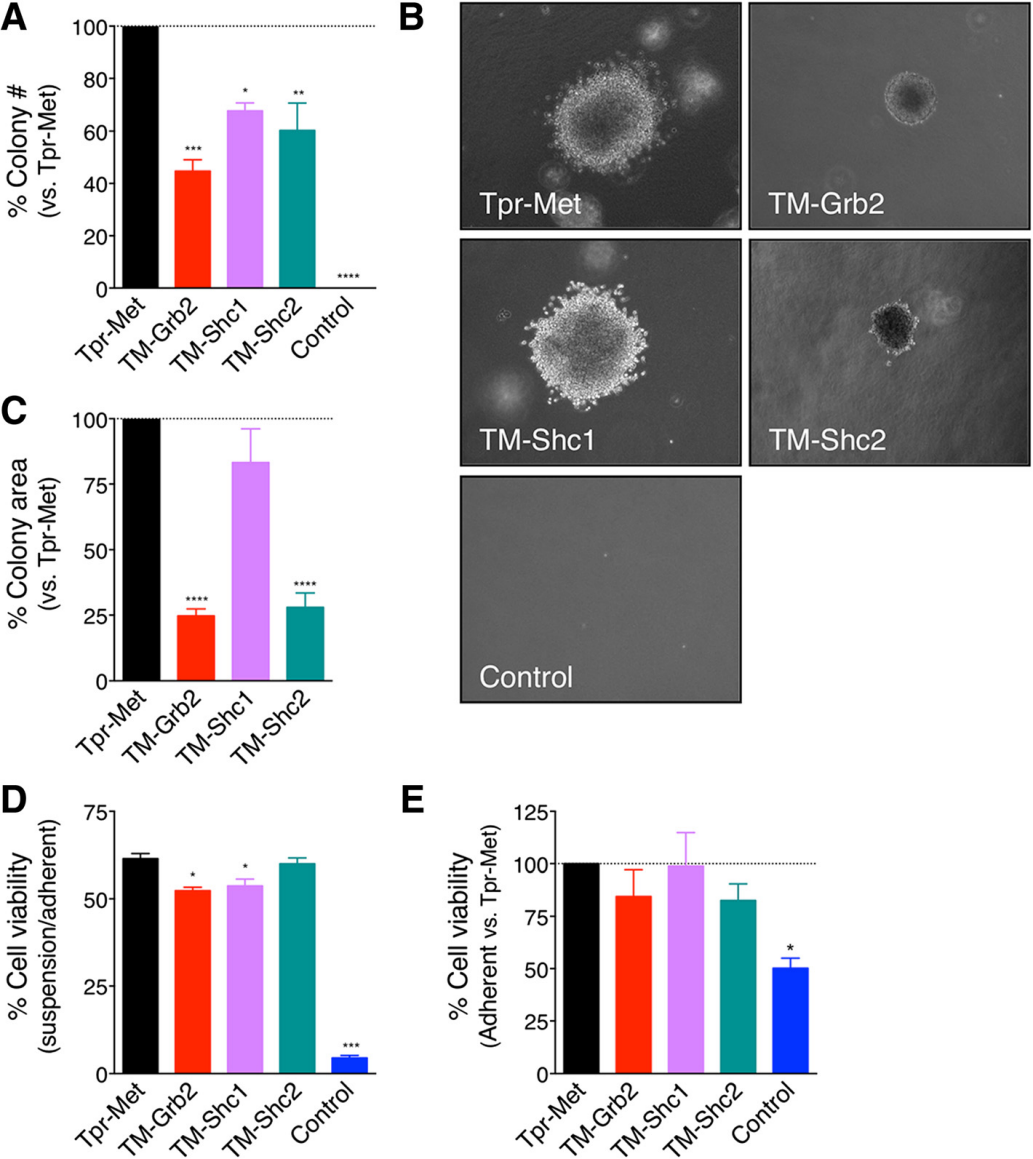
**TM-Grb2, TM-Shc1 and TM-Shc2 oncoproteins induce anchorage-independent growth and anoikis resistance in IEC-6 cells. (A)** The ability of the indicated IEC-6 cell populations to grow in an anchorage-independent manner was tested in soft agar assays. The bar graph represents the average number of colonies formed in soft agar, expressed as percentage ± S.E.M. of those produced by Tpr-Met-IEC-6 cells, from 3 independent experiments performed in triplicate. Tpr-Met-IEC-6 cells, when seeded at a density of 5000 cells in a 6-well plate, were producing an average of 300–500 colonies after ~ 10 days. **(B)** Photographs depict typical morphology of the colonies formed in soft agar. **(C)** The bar graph represents the average size of colonies formed in soft agar, expressed as percentage ± S.E.M. of those produced by Tpr-Met-IEC-6 cells, from 3 independent experiments performed in triplicate. **(D)** Anoikis sensitivity of these IEC-6 cell populations was tested. Cell viability was measured by XTT assays 18 hours after seeding the cells in suspension or adherent conditions. The bar graph shows in percentage the mean ± S.E.M. value of viable cells grown in suspension relative to the adherent ones, calculated from 4 independent experiments performed in triplicate. **(E)** Cell viability in adherent condition, expressed as the percentage mean ± S.E.M. relative to Tpr-Met, is shown.

We next verified whether the oncogenic activation of Grb2- or Shc-dependent signals is sufficient to confer to non-transformed IEC-6 cells the capacity to grow in the absence of anchorage to the extra-cellular matrix (ECM), by performing soft agar growth assays. Consistent with previous findings [5], Control-IEC-6 cells failed to grow in soft agar whereas Tpr-Met-IEC-6 cells grew efficiently, forming numerous large colonies (Figure 3A–C). TM-Grb2-IEC-6, TM-Shc1-IEC-6, and TM-Shc2-IEC-6 cells also formed colonies in soft agar, but with lower efficiencies. While the three IEC-6 cell populations expressing docking-specific Tpr-Met variants formed similar numbers of colonies (Figure 3A), those produced by the TM-Shc1-IEC-6 cells were larger and displayed a different morphology from those produced by the TM-Shc2-IEC-6 and TM-Grb2-IEC-6 cells (Figure 3B and C). Colonies formed by the TM-Shc1-IEC-6 cells were composed mainly of loosely associated cells with apparently limited cell-cell contacts, similar to Tpr-Met-IEC-6 colonies (Figure 3B). By contrast, colonies produced by the TM-Grb2-IEC-6 or TM-Shc2-IEC-6 cells were compact and composed of tightly associated cells (Figure 3B).

Growth in soft agar reflects not only the capacity of cells to proliferate in the absence of ECM attachment, but also to avoid anoikis, a form of apoptosis induced upon loss of matrix attachment [32]. Anoikis assays were performed by monitoring the viability of cells seeded in the absence of serum under adherent and suspension conditions. As expected for normal IECs [33,34], Control-IEC-6 cells displayed anoikis sensitivity under suspension culture conditions (Figure 3D). In contrast, IEC-6 cells transformed by either Tpr-Met, or the Grb2 or Shc docking-specific oncoproteins, displayed potent resistance to anoikis, with more than half surviving under non-adherent conditions. Likewise, the viability of these transformed cells was significantly higher than that of Control-IEC-6 cells when seeded in the absence of serum and under adherent conditions (Figure 3E). Overall, these results indicate that the oncogenic engagement of signals downstream of Grb2 or Shc is sufficient to alleviate the anchorage-dependence of growth, and to reduce sensitivity to growth factor deprivation and to anoikis in IECs.

### Silencing of Grb2 impairs Tpr-Met-induced morphological transformation and anoikis resistance in IECs, but reduced expression of Shc decreases cell growth

**Figure 4:**
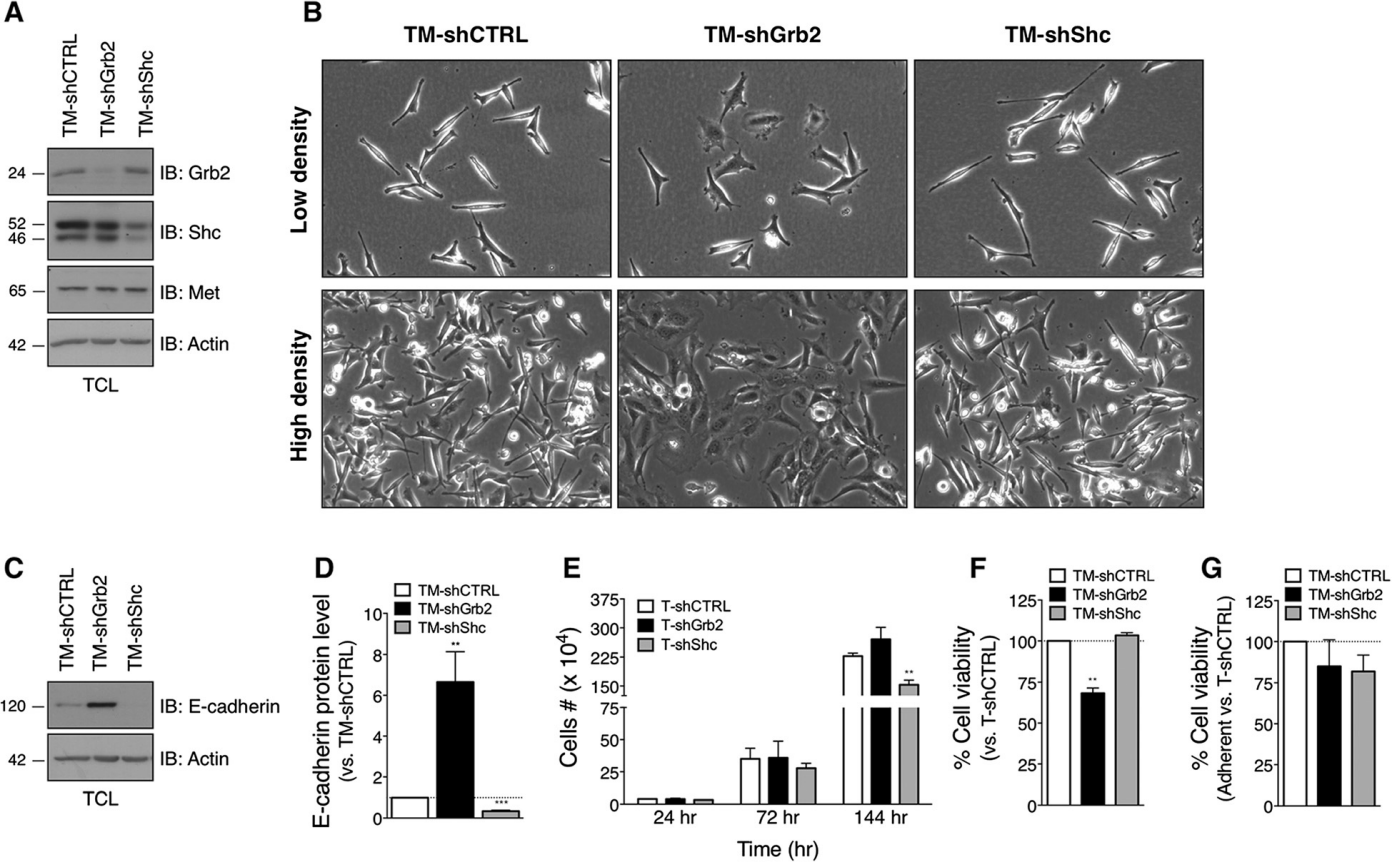
**Silencing of Grb2 in Tpr-Met-IEC-6 cells restores an epithelioid morphology, E-cadherin expression and anoikis sensitivity, while Shc knockdown decreases cell growth. (A)** Protein levels of Grb2, Shc and Tpr-Met were assessed by IB analyses of lysates of serum starved Tpr-Met-IEC-6 cell populations stably expressing shRNA against Grb2 (TM-shGrb2) or Shc (TM-shShc), or a control non-targeting shRNA (TM-shCTRL). Densitometry analyses of IB revealed close to 60% reduction in Grb2 and Shc protein levels in TM-shGrb2 and TM-shShc cells, respectively, when compared to TM-shCTRL. **(B)** Photographs show typical morphology of the indicated shRNA-expressing Tpr-Met-IEC-6 cells when cultured in the presence of serum at either low or high density. **(C)** E-cadherin protein levels were determined by IB analyses in lysates of serum-starved cells. **(D)** Densitometric analysis of E-cadherin protein levels normalized to actin was performed. The bar graph shows the mean ± S.D. fold-change relative to TM-shCTRL cells calculated from at least 3 independent experiments. **(E)** Cell-count assays were performed at the indicated time after seeding the cells under adherent culture conditions and in presence of serum. Bar graph shows the mean number of cells ± S.E.M., from 3 independent experiments performed in triplicate. **(F)** Anoikis sensitivity of the indicated cell populations was evaluated. Cell viability was measured by XTT assays 18 hours after seeding the cells in suspension or adherent conditions. The bar graph shows in percentage the mean ± S.E.M. cell viability in suspension relative to in adherent state, normalized to TM-shCTRL cells calculated from 3 independent experiments performed in triplicate. **(G)** The bar graph shows in percentage the mean ± S.E.M. of cell viability in adherent condition expressed relative to Tpr-Met.

The above results with the docking-specific Tpr-Met-derived variants established that the oncogenic activation of either Grb2 or Shc signaling pathways was each sufficient to promote transformation of IECs, conferring upon them aberrant growth characteristics and resistance to anoikis. We next sought to determine whether signaling pathways downstream of these adaptor proteins were required for the oncogenic potential of Met in IECs. The impact of silencing the expression of Grb2 or Shc on the features of the Tpr-Met-IEC-6 cells was evaluated. Stable populations of Tpr-Met-IEC-6 cells displaying marked and selective knockdown of Grb2 (TM-shGrb2) or Shc (TM-shShc) were generated using shRNAs. As demonstrated by IB analyses (Figure 4A), Grb2 and Shc (all ShcA isoforms) protein levels were selectively reduced by more than 60% in TM-shGrb2 and TM-shShc cell populations, respectively, compared to Tpr-Met-IEC-6 cells expressing a non-targeting shRNA (TM-shCTRL). Protein levels of Tpr-Met and actin remained equivalent amongst all these cell populations. Phase contrast microscopy revealed that the TM-shGrb2 cells exhibited a partial reversal of the transformed morphology, relative to TM-shCTRL cells, characterized by a decrease in cell refractility and an increase in cell spreading (Figure 4B). In contrast, the TM-shShc cells maintained the transformed morphology, and even adopted a slightly more elongated and spindle-shaped appearance than the control TM-shCTRL cells. Concordant with these morphological changes, E-cadherin protein levels were enhanced in TM-shGrb2 cells and reduced in TM-shShc cells, when compared to TM-shCTRL cells (Figure 4C and D).

Growth and survival characteristics of these cells were then evaluated in cell-count and anoikis assays, respectively. While the TM-shGrb2 cells displayed similar growth capacity to TM-shCTRL cells, growth of the TM-shShc cells was observed to decrease in a time-dependent manner, reaching significant inhibition (37.67 ± 5.32%) 3 days after seeding (Figure 4E). The TM-shGrb2 cells displayed enhanced anoikis sensitivity when compared to the TM-shCTRL and TM-shShc cells, between which no difference in survival in suspension was observed (Figure 4F). As shown in Figure 4G, no significant difference in cell viability was observed between the TM-shGrb2, TM-shShc, and TM-shCTRL cells 18 hours following seeding in the absence of serum under adherent conditions (Figure 4G). Together, these results suggest that signals downstream of Grb2 and Shc proteins are required for non-overlapping functions promoted by the oncogenic Met receptor in IECs.

### MEK, but not PI3K inhibitors reduce IEC transformation promoted by the oncogenic engagement of Met, Grb2, or Shc signals

**Figure 5:**
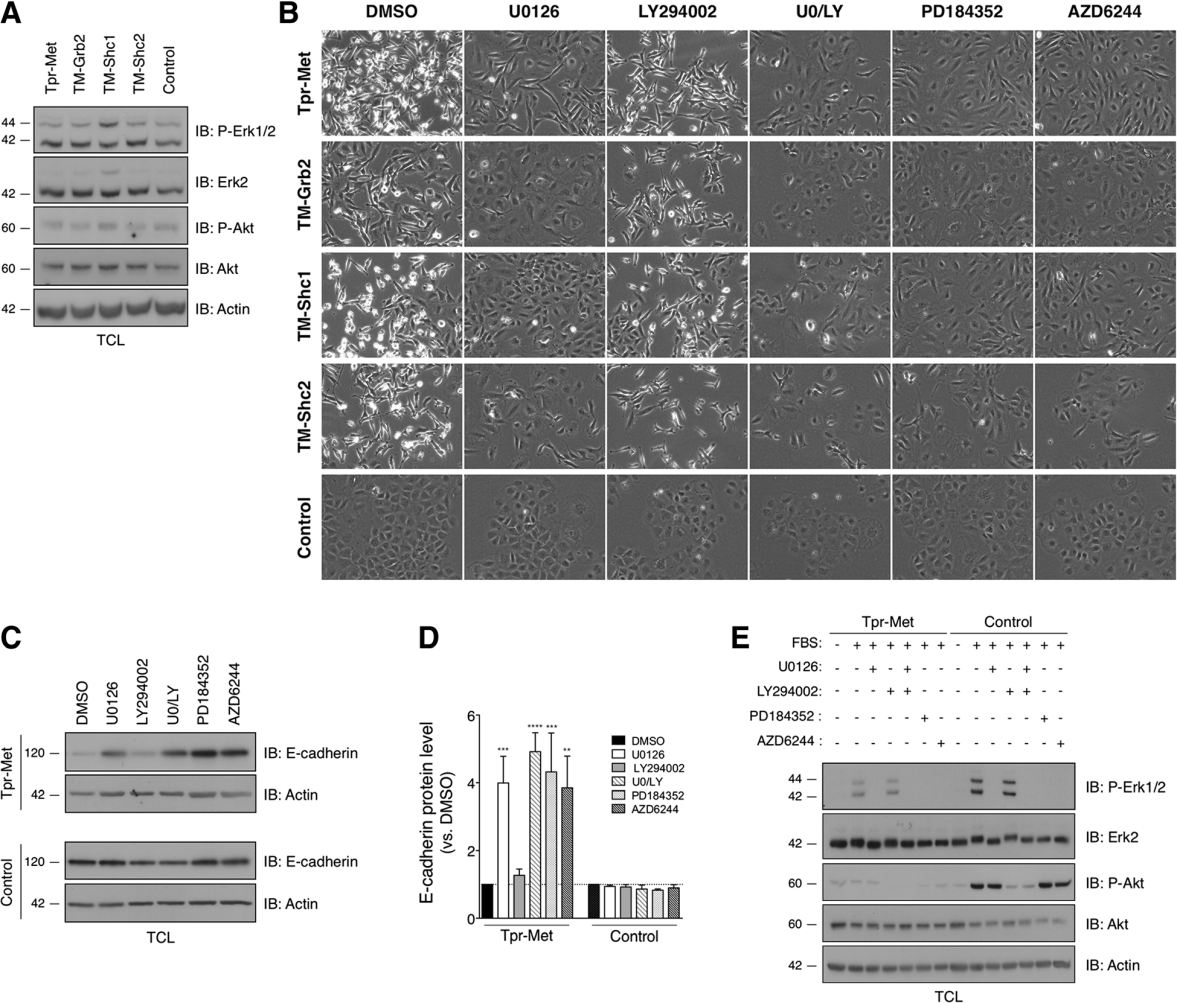
**Inhibitors of MEK1/2, but not of PI3K, inhibits morphological transformation and restores E-cadherin expression in IEC-6 cells transformed by Tpr-Met, TM-Grb2, TM-Shc1 and TM-Shc2 oncoproteins. (A)** Basal phosphorylation and expression levels of Erk1/2 and Akt were assessed by IB analyses of TCL prepared from the indicated serum-starved IEC-6 cells. **(B)** The indicated cells were treated with vehicle (DMSO), or 10 μM of MEK1/2 and/or PI3K inhibitors. Photographs show typical morphologies following 48 hours of treatment. Similar morphological changes were observed after 24 hours of treatment (Additional file 3). **(C)** E-cadherin and actin protein levels were assessed by IB analysis after 48 hours of treatments with DMSO or the indicated inhibitors. **(D)** Relative E-cadherin and actin protein levels were determined by densitometry analysis. The bar graph shows the mean ± S.D. fold-changes in E-cadherin protein levels normalized to that of actin relative to DMSO treated cells. The values were calculated from at least three independent experiments. **(E)** The MEK1/2 and PI3K inhibitors were validated to selectively block serum-induced Erk1/2 and Akt phosphorylation in Tpr-Met-IEC-6 and Control-IEC-6 cells. Serum-starved cells were treated for 1 hour with DMSO or indicated inhibitors, prior to 5 minutes of stimulation with 10% serum. Erk1/2 and Akt phosphorylation and total protein levels were heaevaluated as above. Actin levels were used as a loading control. The results are representative of at least 2 independent experiments.

The Grb2 and Shc adaptor proteins are known to couple RTKs, such as the Met receptor, to the Ras/MAPK and PI3K/Akt signaling pathways [28-30]. The Ras/MAPK and PI3K/Akt pathways are important regulators of RTK-mediated epithelial cell transformation, but their actions are cell type-specific [31]. To define the role of these signaling pathways in IEC transformation, we first compared their activation status in IEC-6 cells transformed by the Tpr-Met, TM-Grb2, TM-Shc1, and TM-Shc2 oncoproteins, to that one in non-transformed Control-IEC-6 cells. The phosphorylation/activation levels of the downstream effectors of MEK and PI3K, Erk1/2 and Akt, respectively, were assessed by IB analyses of lysates prepared from serum-starved IEC-6 cells. Phosphorylation levels of neither Erk1/2 nor Akt were elevated in Tpr-Met-IEC-6, TM-Grb2-IEC-6, TM-Shc1-IEC-6, or TM-Shc2-IEC-6 cells, relative to Control-IEC-6 cells (Figure 5A). Both Erk2 and Akt protein levels were comparable in all IEC-6 cell populations. A similar trend of Erk1/2 and Akt phosphorylation was observed in cells maintained in the presence of serum (data not shown). The lack of evidence for Erk1/2 and Akt activation in IEC-6 cells stably expressing the constitutively activated form of the Met receptor, or the Grb2 and Shc docking-specific oncoproteins, is consistent with both the Ras/MAPK and PI3K/Akt pathways being subject to negative feedback mechanisms [35]. It cannot be explained as merely a cell type–specific event, since similarly stable expression of these oncoproteins in fibroblasts failed to promote Erk1/2 or Akt activation [36] and unpublished observation].

We next investigated whether, even at the low levels observed, MEK or PI3K activities were implicated in the induction of transformation features in IEC-6 cells by the Tpr-Met, TM-Grb2, TM-Shc1, or TM-Shc2 oncoproteins. Cells were treated with vehicle (DMSO), 10 μM U0126 (a MEK1/2 inhibitor), 10 μM LY294002 (a PI3K inhibitor), or a combination of both inhibitors, and their morphology was examined by phase contrast microscopy, following 24 (Additional file 3) and 48 hours of treatment (Figure 5B). Neither individual inhibitor treatment, nor the combination, had an obvious effect on the morphology of the Control-IEC-6 cells. Interestingly, the MEK1/2 inhibitor, but not the PI3K inhibitor, induced a potent reversion of the transformed morphological features of the Tpr-Met-IEC-6, TM-Grb2-IEC-6, TM-Shc1-IEC-6, and TM-Shc2-IEC-6 cells, observed within 24 hours of treatment (Additional file 3) and more striking in appearance after 48 hours (Figure 5B). In the presence of U0126, the MEK1/2 inhibitor, either alone or in combination with the PI3K inhibitor, the formerly transformed Tpr-IEC-6 cells progressively lost their fibroblast-like spindle-shaped morphology, adopted a flatter cobblestone-like appearance, reformed apparent cell-cell contacts and grew again in colonies; much like the non-transformed Control-IEC-6 cells.

Concordant with this restoration of epithelioid-like morphological features in IEC-6 cells transformed by the oncogenic Tpr-Met and its derived variants, treatment with U0126 also induced an increase in E-cadherin protein levels (Figure 5C and D, and Additional file 3). By contrast, none of the inhibitor treatments affected E-cadherin protein levels in the Control-IEC-6 cells (Figure 5C and D). Notably, reversion of the transformed phenotype and E-cadherin up-regulation were also promoted in transformed IEC-6 cell populations by treatment with 10 μM AZD6244 or PD184352, two additional pharmacological inhibitors of MEK1/2 (Figure 5B and C, and Additional file 3). Furthermore, these observations could not be attributed to the cytosolic localization of the Tpr-Met oncoprotein, since both the morphological transformation and the E-cadherin down-regulation induced by a cell surface-localized active chimeric colony stimulating factor 1 (CSF)-Met receptor [37], were reverted in a similar manner upon the inhibition of MEK1/2 activity, but not of PI3K activity (Additional file 3).

Since Erk1/2 and Akt activities remained at basal levels in transformed IEC-6 cell populations, the efficacy of these pharmacological inhibitors was evaluated by testing their ability to suppress serum-induced Erk1/2 and Akt phosphorylation. Serum-starved Tpr-Met and control IEC-6 cell populations were treated for 1 hour with DMSO or inhibitors, followed by 5 minutes of stimulation with 10% serum. A robust phosphorylation of Erk1/2 and Akt proteins was seen upon serum stimulation of Control-IEC-6 cells (Figure 5E). In sharp contrast, although the levels of Erk2 and Akt proteins were equivalent for each cell population, serum-induced Erk1/2 and Akt activation were severely attenuated in the Tpr-Met-IEC-6 cells (Figure 5E). This further substantiates the conclusion that sustained activation of Met signaling pathways, such as those downstream of Grb2 and Shc, can activate negative feedback control of both the Erk1/2 and Akt pathways in IECs. Importantly, the MEK1/2 and PI3K inhibitors were confirmed to efficiently suppress serum-induced Erk1/2 and Akt phosphorylation, respectively (Figure 5E). Although the potential off-target effects of these inhibitors cannot be excluded in these experiments, our results suggest that the morphological transformation induced by the oncogenic Met receptor, and driven by the constitutive activation of Grb2 and Shc signals in IECs, relies, at least in part, on the activation of the Ras/MAPK pathway, but not on PI3K signaling.

### The growth promoting effect of oncogenic Met in IECs is blocked by MEK inhibition, but anoikis sensitivity is restored by concomitant treatment with MEK and PI3K inhibitors

**Figure 6:**
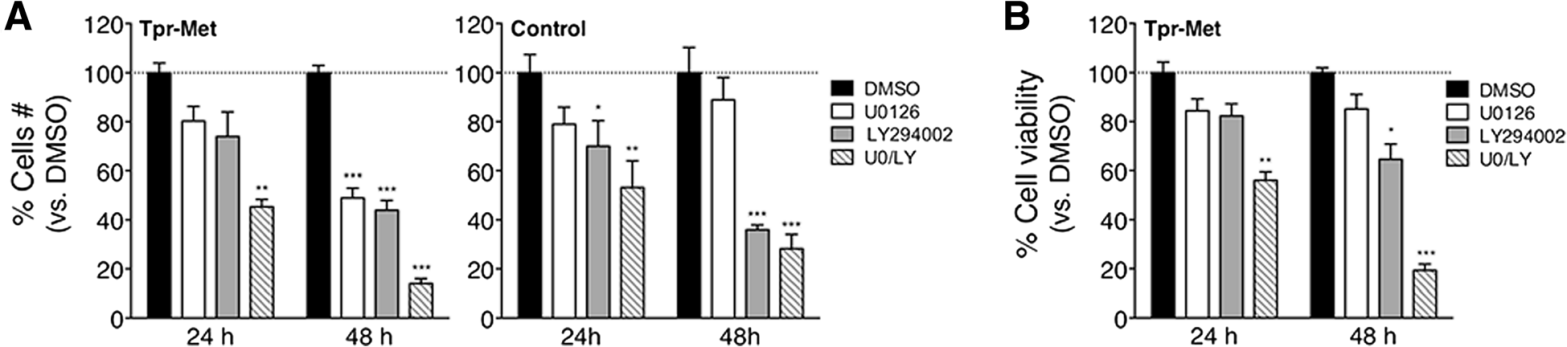
**Tpr-Met-mediated enhanced growth in IECs is reduced by inactivation of MEK1/2, and resistance to anoikis is restored by dual inactivation of MEK1/2 and PI3K. (A)** Cell-count assays were performed with Tpr-Met and Control-IEC-6 cells cultured under adherent conditions and in presence of serum, after 24 and 48 hours of treatment with vehicle (DMSO), 10 μM of the MEK1/2 or PI3K inhibitor, or a combination of both inhibitors. The bar graph shows as percentage the mean number of cells ± S.E.M. relative to DMSO-treated cells. **(B)** Anoikis assays were conducted 24 and 48 hours after seeding Tpr-Met-IEC-6 cells in suspension, and concurrently in the presence of the vehicle or indicated inhibitors. The bar graph represents in percentage the mean value ± S.E.M. of cell viability normalized to DMSO-treated cells, calculated from 3 independent experiments performed in triplicate.

We next investigated whether the growth capacity promoted by the oncogenic Met in IECs required MEK or PI3K activity. Cell-count assays were performed with Tpr-Met and Control-IEC-6 cells that were treated for 24 or 48 hours with vehicle, U0126 or LY294002 inhibitor, or a combination of both inhibitors. As anticipated, treatment with DMSO did not significantly impact cell growth, even after 48 hours. Treatment with LY294002 exerted similar inhibitory effects upon the growth of both the Tpr-Met-transformed and control IEC-6 cells, reducing the number of cells by 44% and 36%, respectively relative to DMSO-treated cells, after 48 hours (Figure 6A). In contrast, in the presence of U0126, the growth of Tpr-Met-IEC-6 cells was significantly attenuated but not that of Control-IEC-6 cells (Figure 6A). Furthermore, while co-treatment with U0126 failed to potentiate the growth inhibiting effect of LY294002 in Control-IEC-6 cells, the growth of Tpr-Met-IEC-6 cells was further reduced in a time-dependent manner upon exposure to both inhibitors (Figure 6A).

The impact of inhibiting MEK1/2 or PI3K activity on the viability of Tpr-Met-IEC-6 cells in suspension was also evaluated. In these anoikis assays, vehicle or the indicated inhibitors were added when cells were seeded and cell viability was measured 24 and 48 hours later. Treatment with the PI3K inhibitor restored anoikis sensitivity to the Tpr-Met-IEC-6 cells in a time-dependent manner, reducing their viability by close to 45% relative to DMSO-treated cells after 48 hours (Figure 6B). The MEK1/2 inhibitor, on the other hand, did not substantially impact the viability of the Tpr-Met-IEC-6 cells grown in suspension. Nonetheless, U0126 treatment did elicit a marked synergistic effect on LY294002-induced anoikis, reducing Tpr-Met-IEC-6 cell viability to levels well below those seen upon treatment with the PI3K inhibitor alone (Figure 6B). These results suggest that Tpr-Met-mediated growth promoting effects observed in IECs implicate MEK-dependent mechanisms, but that the anoikis resistance involves the integration of both MEK- and PI3K-dependent signaling pathways.

## Discussion

As normal intestinal epithelial cells become cancerous, they gain the ability to grow aberrantly by evading normal growth-inhibiting and death signals, as well as the ability to invade tissue [38]. Experimental and clinical studies suggest that the deregulation of RTKs plays a critical role in the etiology and progression of human CRC [2-4]. These studies highlight the ability of RTKs to induce biological characteristics linked with tumorigenesis and metastatic progression [1,38]. However, the proximal signaling molecules recruited by RTKs have not yet been assigned individual contributions to the neoplastic transformation of normal IECs. In this study, Met-derived docking-specific variants were used to define the cancer properties induced upon the RTK-mediated engagement of the Grb2 or Shc adaptor proteins in IECs. Our results show, for the first time in a non-transformed IEC model, that the sustained activation of signals downstream of either Grb2 or Shc alone is sufficient to promote morphological transformation, E-cadherin down-regulation, enhanced cell growth, loss of contact inhibition of growth, the acquisition of anchorage-independence of growth, and anoikis resistance (Figures 1, 2, 3). These oncogenic features are prerequisites for the progression of epithelial-derived cancers, favoring the survival and growth of cancerous cells in the matrix-poor, disorganized extracellular environments often found in primary tumors, and in systemic circulation, facilitating metastasis [32,38]. Thus, our results provide novel evidence for a causal role of RTK-linked Grb2 and Shc signaling pathways in important and common phenotypic features of neoplastic transformation of IECs and metastatic CRCs.

Expression of the cell adhesion molecule, E-cadherin, is typically depleted from cell-cell contacts in epithelial cancer cells, or even shut down altogether [31]. Cellular loss of E-cadherin leads to dissolution of adherens junctions and to a reduction in cell-cell contacts, facilitating migration and invasion, both of which are key processes for metastatic dissemination of epithelial tumor cells. Notably, an inverse correlation exists between E-cadherin levels in human CRC specimens and cancer grade, invasiveness of tumor phenotype, metastatic disease progression, and poor patient prognosis [39]. Multiple mechanisms have been identified that promote E-cadherin down-regulation in epithelial cells, in response to different stimuli and/or in different cell types. These include transcriptional silencing via deregulation of transcription factors (Snail, Twist, and Zeb) or promoter hyper-methylation, and internalization followed by subsequent lysosomal degradation mediated by post-translational modifications [31,40]. E-cadherin protein levels were further reduced in IEC-6 cells expressing the Shc docking-specific oncoproteins than those transformed by TM-Grb2 or Tpr-Met (Figure 1). Differential expression levels of these oncoproteins cannot fully account for this response, therefore suggesting that Grb2 and Shc might mediate E-cadherin down-regulation by distinct mechanisms. However, E-cadherin levels and an epithelial non-transformed typical morphology were restored upon pharmacological inhibition of MEK1/2 but not of PI3K, activities in IECs transformed by these oncoproteins (Figure 5 and Additional file 3). These findings suggest that the engagement of Grb2 and Shc, like Met, promotes these oncogenic features through shared signaling pathways. Furthermore, E-cadherin repression induced by oncogenic Met signaling pathways in IECs, like that driven by Grb2 and Shc, was associated with an up-regulation of the repressors of E-cadherin transcription *Snail2*, *Twist1,* or *Twist2*, but not of *Snail1* or *Zeb1* (Additional file 2). Future studies will be needed to define the shared and distinct mechanisms that result in E-cadherin down-regulation in IECs downstream of Grb2 or Shc. Nonetheless, our results suggest that these adaptor proteins are important integrators of signals leading to the neoplastic transformation and E-cadherin dysfunction in CRC harboring the deregulated Met receptor, and most likely other RTKs.

A role for deregulated RTKs in conferring anoikis resistance to IECs through cross-talk with cell adhesion receptors, is well-established [32]. While transforming growth factor-α (TGF-α) stimulation protects IECs from anoikis [41], CRC cells that express high levels of EGF/TGF-α evade anoikis through autocrine stimulation of the EGFR [33]. However, the importance of Grb2 or Shc functions in anoikis resistance in IECs has not been addressed. Furthermore, of the very few investigations of the HGF/Met receptor axis in anoikis control and cancer, a single study has reported a key role for the Met receptor in CRC cells [42]. Activation of the Met receptor has been reported to prevent anoikis in human colon, ovarian, pancreatic, and head and neck carcinoma cell lines by mechanisms dependent on PI3K, but with varied requirement for MEK1/2 [42-45]. Herein, we have shown that while individual inhibition of PI3K activity, but not of MEK1/2, partially restored anoikis sensitivity in Tpr-Met-IEC-6 cells, concurrent inhibition of these pathways exerted a synergistic effect (Figure 6). Notably, anoikis resistance driven by the oncogenic Met receptor in IECs is partly dependent on Grb2 functions, whereas Shc functions appear dispensable (Figure 4). Our findings suggest a model whereby deregulation of Met might promote anoikis resistance in CRC cells, through the integration of both MEK and PI3K signaling pathways, and likely involving the engagement of Grb2. Collectively, our results provide novel evidence that signaling pathways engaged by deregulated RTKs in CRC, including those reliant on Grb2 or Shc, may represent important regulators of anoikis resistance in IECs, a process of outmost relevance in cancer metastasis.

We show that oncogenic Met receptor-dependent signals, like those activated downstream of Grb2 and Shc, trigger negative feedback upon the Ras/MAPK and PI3K/Akt pathways in IECs, restricting Erk and Akt activation (Figure 5 and Additional file 3). Although somewhat controversial, some studies suggest that Erk hyperactivation may only occur in a small subset of CRC tumors, and that Erk activity is more often elevated in adjacent normal tissue [46-48]. Also, Erk activity in human CRC tumors appears to be a poor predictor of activating *K-RAS* mutation status and of the effectiveness of MEK inhibition [48-50]. We report the inhibition of the IEC transformation and E-cadherin down-regulation induced by each of our oncoproteins by inhibitors of MEK activity, but not of PI3K activity (Figure 5 and Additional file 3). Cell growth and anoikis resistance evoked by Tpr-Met, on the other hand, was blocked by concomitant treatment with MEK and PI3K inhibitors (Figure 6). Thus, our findings suggest that while growth factor stimulation is linked to the activation of the Ras/MAPK and PI3K/Akt pathways, in part through Grb2 and Shc, Erk or Akt activity levels in CRC may not reliably predict the extent of RTK deregulation, nor the sensitivity to therapies targeting them.

The Tpr-Met and the Grb2- and Shc-specific docking oncoproteins are all predicted to promote cancer features in IECs by engaging similar signaling pathways. Indeed, they share the ability to complex with the Gab1 scaffolding protein. While binding to TM-Grb2 and TM-Shc oncoproteins by Grb2-dependent mechanisms, Gab1 also interacts directly with the Met receptor [8,20,51]. Notably, Gab1 has been shown to be required for Erk and Akt activation, and many oncogenic functions downstream of Met, and the Grb2- and Shc-docking oncoproteins in fibroblast, MDCK epithelial, and *Xenopus* cell models [20,28]. Thus, it may be that Gab1 provides a platform for the integration of Ras/MAPK and PI3K/Akt positive and negative signals downstream of these oncoproteins and relevant to their oncogenic functions in IECs. However, Tpr-Met-IEC-6 cells were observed to display stronger transformed phenotypes than cells expressing the Grb2 or Shc-binding variants oncoproteins, for example in focus-formation and growth in soft agar (Figures 2 and 3). This suggests that Tpr-Met may activate pathways not engaged by the Tpr-Met Shc or Tpr-Met Grb2 oncoproteins. Furthermore, it is now acknowledged that Shc, by interacting with proteins other than Grb2 such as IQGAP1, Crk and Sgk269, can promote Grb2-independent pathways and functions [13,17-19], underscoring the complexity of the cellular networks that these adaptor proteins can engage downstream of RTKs. It is therefore anticipated that the Grb2- and Shc-specific docking oncoproteins, and Tpr-Met may prove, upon further analyses, to mediate distinct signaling pathways, and therefore specific cancer processes in IECs.

Therapies targeting RTKs are recognized as a promising avenue for the treatment of cancer, but the clinical benefits observed with these agents have so far been modest. As typified by EGFR-targeted therapies for metastatic CRC (e.g.: panitumumab and cetuximab), this modest response is attributed to the innate and acquired proficiency of cancer cells to escape EGFR inhibition by engaging alternative oncogenic signals [2,3,52]. Multiple mechanisms of resistance have been proposed, including the manifest heterogeneity of RTKs being deregulated in CRC cells [52,53]. Notably, activation of the Met/HGF receptor axis is emerging as an important mechanism of resistance to drugs targeting oncogenic kinases in human cancers, including CRC, while concurrent inhibition of multiple RTKs in CRC cells seems to offer better therapeutic effects than targeting a specific RTK [53-60]. An alternative way to achieve similar outcomes might be offered by targeting RTK-proximal signaling effectors engaged by all, or at least several RTKs, particularly those regulating biological processes critical for the initiation and/or progression of CRCs. In this regard, we show that although oncogenic engagement of Grb2 or Shc triggers redundant cancer properties in IECs (Figures 1, 2, 3), these adaptor proteins were proven, through analysis of the impact of their silencing in Tpr-Met-transformed IECs, to be necessary for non-overlapping functions (Figure 4). The silencing of Shc in Tpr-Met-IEC-6 cells was demonstrated to partly reduce cell growth without impacting anoikis resistance, but slightly increasing transformation and E-cadherin down-regulation. These results indicate that the Met receptor has the intrinsic capacity to circumvent the loss of Shc functions by engaging alternative oncogenic signals, likely involving the adaptor proteins Grb2, Gab1, or others effectors. Conversely, inhibition of Grb2 functions restored normal non-transformed epithelial morphology, E-cadherin expression, and anoikis sensitivity in these same Met-transformed IECs. Incidentally, Grb2 SH2 domain-binding antagonists were shown *in vitro* to block HGF-induced migration and invasion in MDCK epithelial cells*,* metastasis formation of melanoma and prostate cancer cells *in vivo*, and the motility of human SW620 CRC cells in wound-healing *in vitro* assays [61-63]. Considering these observations, with our current findings, we suggest the targeting of Grb2 signaling in CRC, particularly in the context of deregulated Met, as a potentially effective therapeutic strategy to reduce CRC metastasis.

## Conclusions

The design of novel CRC therapies is contingent on a better understanding of the mechanisms underlying the ability of deregulated RTKs to relay downstream signaling pathways that convey oncogenic properties in normal IECs. In this study, we provide evidence that Met-driven oncogenic activation of Grb2 or Shc signaling leads to the neoplastic transformation of normal IECs and induces multiple redundant hallmarks of cancer in these cells. Sustained engagement of Grb2 and Shc in IECs was also identified to evoke negative feedback control of the Ras/MAPK and PI3K/Akt pathways, limiting their degree of activation, however these pathways seem to remain critical to oncogenic functions. Notably, our data also illustrate the functional non-redundancy of Grb2 and Shc downstream of Met, and suggest that Grb2 might represent a promising target for the design of novel therapies for CRC harboring deregulated Met, and possibly other RTKs.

## Competing interests

The authors declare that they have no competing interests.

## Authors’ contributions

VP contributed to the conception of the study, performed cell and molecular studies, and drafted the manuscript. ML carried out part of the anoikis and pharmacological studies. JB generated and carried out the initial characterization of the IEC populations expressing the Tpr-Met and docking-specific variants. PHV participated in drafting the manuscript. CS coordinated all aspects of the study and participated in the writing of the manuscript. All authors read and approved the final manuscript.

## Pre-publication history

The pre-publication history for this paper can be accessed here:

http://www.biomedcentral.com/1471-2407/14/240/prepub

## Supplementary Material

Additional file 1 TM-Grb2, TM-Shc1, and TM-Shc2 oncoproteins display the expected docking specificity when expressed in IEC-6 cells. Click here for file

Additional file 2 Oncogenic Met, Grb2, and Shc signaling pathways alter the expression of critical E-cadherin transcriptional repressors. Click here for file

Additional file 3 IEC transformation induced by oncogenic Met, Grb2, and Shc signaling requires MEK but not PI3K, activity. Click here for file
