# Supplementary material for: Met receptor-induced Grb2 or Shc signals both promote transformation of intestinal epithelial cells, albeit they are required for distinct oncogenic functions
Source: BMC Cancer. 2014 Apr 4;14:240. doi: 10.1186/1471-2407-14-240 (PMC4234027; doi:10.1186/1471-2407-14-240)
Supplement: Additional file 1 — TM-Grb2, TM-Shc1, and TM-Shc2 oncoproteins display the expected docking specificity when expressed in IEC-6 cells. [file 1471-2407-14-240-S1.pdf]

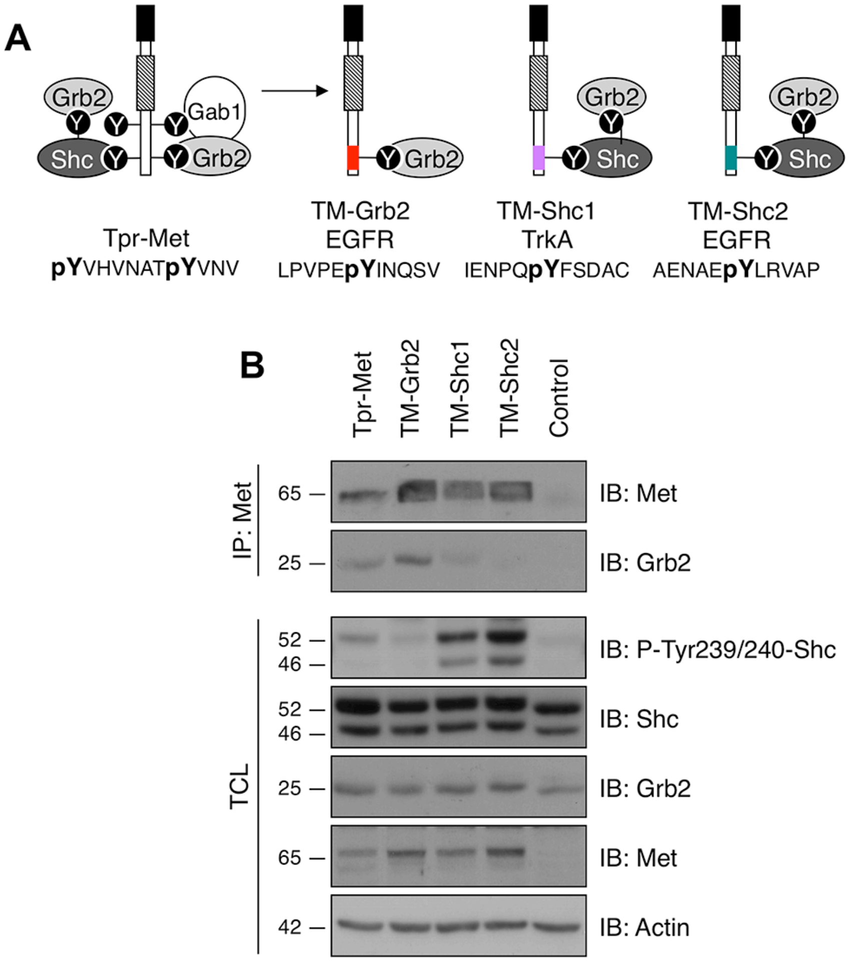

Additional file 1 – TM-Grb2, TM-Shc1, and TM-Shc2 oncoproteins display the expected docking specificity when expressed in IEC-6 cells. (**A**) Schematic representation of the Tpr-Met variant oncoproteins specific for the direct recruitment of either Grb2 or Shc. The amino acids surrounding Y489 in Tpr-Met, and those constituting the binding motifs inserted to produce the Grb2 (TM-Grb2) or Shc (TM-Shc1 and TM-Shc2) docking-specific variants, derived from the EGFR and the TrkA receptor, are shown. (**B**) Expression levels of Tpr-Met, TM-Grb2, TM-Shc1, and TM-Shc2 in IEC-6 cells, and their selectivity engaging Grb2 or Shc proteins. Variant Tpr-Met oncoproteins were immunoprecipitated (IP) from the indicated total cell lysates with an antibody raised against the human Met receptor (IP: Met, top two panels). The amount of Grb2 and Tpr-Met oncoproteins recovered was then evaluated by immunoblot (IB) analysis. As anticipated, coimmunoprecipitation (co-IP) of Grb2 protein was primarily observed with the TM-Grb2 and Tpr-Met oncoproteins which both contain a high affinity pYxNx Grb2 consensus-binding site. Low levels of association between Grb2 and the oncoproteins designed for recruiting Shc were also detected, reflecting their ability to recruit Grb2 through indirect mechanisms [8, 9, 15, 16, 20]. As previously observed, detection of the Shc proteins in these co-IPs was precluded by the relatively low levels of Tpr-Met variant and Shc proteins present in these stable cell populations. However, consistent with their docking selectivity, robust Tyr phosphorylation of Shc proteins in total cell lysates of IEC-6 populations expressing either the TM-Shc1 or TM-Shc2 oncoproteins was demonstrated by IB analysis with a phosphoTyr-Shc-specific antibody. In comparison, the extent of Shc protein Tyr phosphorylation in cells expressing Tpr-Met was reduced, but above levels detected in either Control-IEC-6 cells or those expressing the TM-Grb2 oncoprotein.
