# Supplementary material for: Met receptor-induced Grb2 or Shc signals both promote transformation of intestinal epithelial cells, albeit they are required for distinct oncogenic functions
Source: BMC Cancer. 2014 Apr 4;14:240. doi: 10.1186/1471-2407-14-240 (PMC4234027; doi:10.1186/1471-2407-14-240)
Supplement: Additional file 2 — Oncogenic Met, Grb2, and Shc signaling pathways alter the expression of critical E-cadherin transcriptional repressors. [file 1471-2407-14-240-S2.pdf]

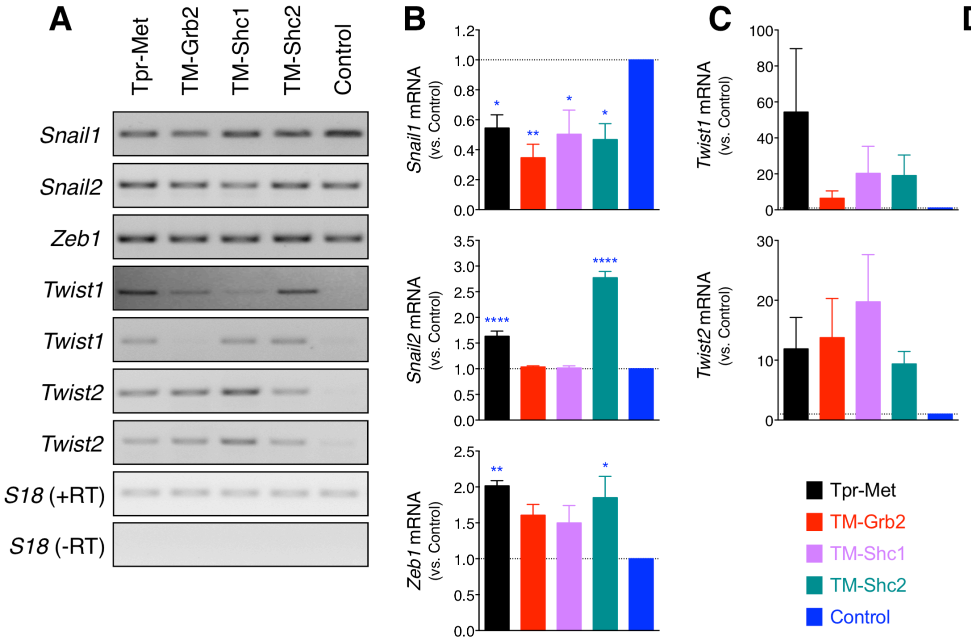

D

Target gene	Primer Sequence 5' - 3'
Cdh1	Fwd GAGACAGGCTGGCTGAAAGT<br>Rev TCACGGAGGTTCCTGGAAGA
Cdh1	Fwd TTCAACCCAAGCACGTACCA<br>Rev CAGAATGCCCTCGTTGGTCT
Snail1	Fwd ATGAGGACAGTGGCAAAAGC<br>Rev TCGGATGTGCATCTTCAGAG
Snail1	Fwd GCGAGCAGAGTTGTCTACCG<br>Rev CTGGAAGGTGAACTCCACACA
Snail2	Fwd GCACTGTGATGCCCAGTCTA<br>Rev CAGTGAGGGCAAGAGAAAGG
Snail2	Fwd TGCCCAGGCTAGGAAATCGT<br>Rev CTTTTCCCCAGTGTGTGTTCT
Zeb1	Fwd GCCAAATGGAAACCAGGATGA<br>Rev TACAGATTCCGCACTCGTGG
Twist1	Fwd GTCATGGCCAACGTGCGGGA<br>Rev CCAGGCCCCCTCCATCCTCC
Twist2	Fwd CCTTCTCCGTGTGGCGCATG<br>Rev GTCCCCTGCCGCTGTTGTCC
S18	Fwd TTGTTGGTTTTCGGAACTGAGG<br>Rev GGCAAATGCTTTCGCTCTGG
TBP	Fwd AGCCTTCCACCTTATGCTCA<br>Rev TGTGTGGGCTGCTGAGATGT
TBP	Fwd CCAAGTGTGAGCCTCTCCAA<br>Rev CAGTGATGTGGGGACAAAACG
Pum1	Fwd CCGACCAGACCCTCCCTATCCT<br>Rev TGCTGACTCAATACAAGTACATTGCCTC
Rpl19	Fwd TGTGGTAAAAAGAAGGTGTGGTTGGA<br>Rev GCCAAGGTGTTCTTCCGGC

Additional file 2 – Oncogenic Met, Grb2, and Shc signaling pathways alter the expression of critical E-cadherin transcriptional repressors. E-cadherin mRNA and protein levels were demonstrated to be further reduced in IEC-6 cells expressing the Grb2 or Shc docking-specific oncoproteins, compared to those transformed by Tpr-Met (Figure 1). Thus, the ability of these oncoproteins to alter the expression of E-cadherin transcriptional repressors was evaluated. (**A**) The mRNA levels of *Snail1*, *Snail2*, *Zeb1*, *Twist1,* and *Twist2* were analyzed by semi-quantitative RT-PCR assays performed with total RNA prepared from the indicated serum-starved cells. The *S18* mRNA level is shown as a loading control. (**B**) Relative expression levels of *Snail1*, *Snail2,* and *Zeb1* mRNAs were evaluated by quantitative RT-PCR analyses. The bar graph presents the mean fold-change (± S.E.M.) of the indicated mRNA levels relative to Control-IEC-6 cells, from three independent experiments performed in duplicate. The *TATA-binding protein* (*TBP*), *pumilio RNA-binding family member 1* (*Pum1*), and *ribosomal protein L19* (*Rpl19*) mRNA levels were used for normalization. The levels of *Snail1* mRNA were significantly reduced in all transformed IEC-6 cells (>4-fold relative to Control-IEC-6 cells). The expression of *Snail2* was slightly, but significantly, elevated in Tpr-Met and TM-Shc2-IEC-6 cells, but not in cells transformed by the TM-Grb2 or TM-Shc1 oncoproteins. A slight increase in *Zeb1* mRNA was observed in all transformed IECs when compared to control cells but did reach significance only in Tpr-Met and TM-Shc2 IEC-cells. The basal mRNA levels of *Twist1* and *Twist2* were too low in the Control-IEC-6 cells for reliable quantification by real-time RT-PCR methods. Thus, relative expression levels of *Twist1* and *Twist2* mRNAs were determined by performing semi-quantitative RT-PCR assays, with gel-based densitometric quantification of amplification products. The bar graph shows the mean fold-change (± S.D.) in *Twist1 or Twist2* mRNA levels relative to Control-IEC-6 cells normalized to *S18* mRNA levels, from three independent experiments. *Twist1* and *Twist2* mRNA levels were noticeably elevated in cells transformed by Tpr-Met, and the Grb2 and Shc docking-specific oncoproteins, but the extent of up-regulation of these two genes was highly variable between multiple assays. (**D**) Primer sequences for these RT-PCR analyses are listed. Overall, these data suggest that a combined up-regulation of *Snail2*, *Twist1* or *Twist2* mRNAs, but not of *Snail1* and *Zeb1,* underlies the down-regulation of E-cadherin induced by Met oncogenic signaling pathways and those of Grb2 and Shc in IECs.
