# Supplementary material for: Met receptor-induced Grb2 or Shc signals both promote transformation of intestinal epithelial cells, albeit they are required for distinct oncogenic functions
Source: BMC Cancer. 2014 Apr 4;14:240. doi: 10.1186/1471-2407-14-240 (PMC4234027; doi:10.1186/1471-2407-14-240)
Supplement: Additional file 3 — IEC transformation induced by oncogenic Met, Grb2, and Shc signaling requires MEK but not PI3K, activity. [file 1471-2407-14-240-S3.pdf]

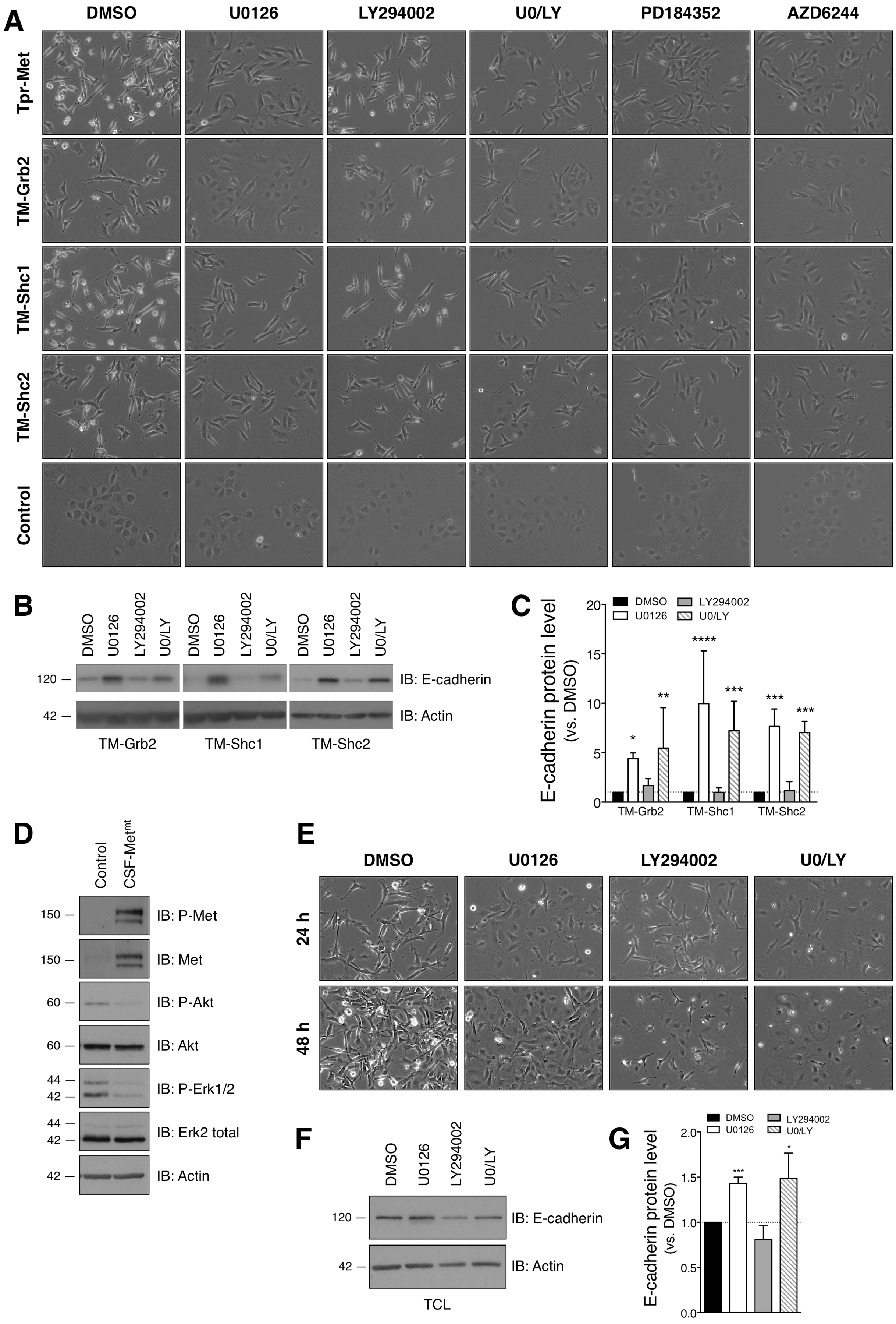
A
DMSO
U0126
LY294002
U0/LY
PD184352
AZD6244
Tpr-Met
TM-Grb2
TM-Shc1
TM-Shc2
Control
B
DMSO
U0126
LY294002
U0/LY
120 —
42 —
IB: E-cadherin
IB: Actin
TM-Grb2
TM-Shc1
TM-Shc2
C
DMSO
U0126
LY294002
U0/LY
E-cadherin protein level
(vs. DMSO)
20
15
10
5
0
*
**
****
***
***
***
TM-Grb2
TM-Shc1
TM-Shc2
D
Control
CSF-Met[mt]
150 —
IB: P-Met
150 —
IB: Met
60 —
IB: P-Akt
60 —
IB: Akt
44 —
42 —
IB: P-Erk1/2
44 —
42 —
IB: Erk2 total
42 —
IB: Actin
E
DMSO
U0126
LY294002
U0/LY
24 h
48 h
F
DMSO
U0126
LY294002
U0/LY
120 —
IB: E-cadherin
42 —
IB: Actin
TCL
G
DMSO
U0126
LY294002
U0/LY
E-cadherin protein level
(vs. DMSO)
2.0
1.5
1.0
0.5
0.0
***
*

Additional file 3 – IEC transformation induced by oncogenic Met, Grb2, and Shc signaling requires MEK, but not PI3K, activity. (**A**) Cells were treated with vehicle (DMSO) or 10 μM selective inhibitors of MEK1/2 (U0126, PD184352, or AZD6244) or PI3K (LY294002). Photographs depict typical cell morphologies following 24 hours of treatment. Cell morphology alterations resulting from 48 hours of treatment are shown in Figure 5. (**B**) E-cadherin and actin protein levels in TM-Grb2, TM-Shc1, and TM-Shc2 cells were assessed by IB analyses following 48 hours of treatments with DMSO or the indicated inhibitors. (**C**) Relative E-cadherin and actin protein levels were determined by densitometry analyses. The bar graph presents the mean fold-changes (± S.D.) in E-cadherin protein levels normalized to that of actin, relative to DMSO treated cells. Values were calculated from at least three independent experiments. The analysis of E-cadherin protein levels in Tpr-Met and control IEC-6 cells are shown in Figure 5. (**D-G**) A constitutively active cell-surface Met receptor mutant promotes morphological transformation of IEC-6 cells by MEK1/2-dependent pathways. As Tpr-Met is a cytosolic oncoprotein, we sought to determine whether oncogenic activation of Met signaling at the cell surface could induce morphological transformation of IECs in a similar manner. Stable IEC-6 cell lines expressing the CSF-Met^{mt} were generated. This cell-surface chimeric Met receptor mutant is composed of the extracellular domain of the human colony stimulating factor-1 receptor fused to the Met transmembrane and intracellular domains. The Y1003F and M1268T substitutions in the Met intracellular domain reduce, respectively, Met degradation by preventing c-Cbl E3 ubiquitin ligase binding and enhance receptor kinase activity. (**D**) Much like Tpr-Met, CSF-Met^{mt} is expressed as a tyrosine phosphorylated protein in IEC-6 cells, as determined by IB analyses of TCLs prepared from serum-starved cells. The phosphorylation levels of neither Erk1/2 nor Akt were elevated in the CSF-Met^{mt}-expressing IEC-6 cells, relative to control cells. Total levels of these proteins were also comparable. (**E**) Photographs demonstrate that the CSF-Met^{mt}-expressing IEC-6 cells were morphologically transformed, and that treatment with the MEK1/2 inhibitor (U0126) , but not with a PI3K inhibitor (LY294002), promoted reversion of the transformed morphological features. (**F**) Restoration of epithelioid-like morphological features by the MEK inhibition in CSF-Met^{mt} IEC-6 cells was concurrent with an increase in E-cadherin protein levels, evaluated by IB analyses. (**G**) Densitometric analyses of E-cadherin protein levels were performed. The bar graph presents the mean fold-change (± S.D.) in E-cadherin protein levels normalized to that of actin, relative to DMSO treated cells. The values were calculated from at least three independent experiments.
